# DiNAR: revealing hidden patterns of plant signalling dynamics using Differential Network Analysis in R

**DOI:** 10.1186/s13007-018-0345-0

**Published:** 2018-08-30

**Authors:** Maja Zagorščak, Andrej Blejec, Živa Ramšak, Marko Petek, Tjaša Stare, Kristina Gruden

**Affiliations:** 1 Department of Biotechnology and Systems Biology, National Institute of Biology, Večna pot 111, 1000 Ljubljana, Slovenia; 2 Department of Organisms and Ecosystems Research, National Institute of Biology, Večna pot 111, 1000 Ljubljana, Slovenia

**Keywords:** Biological networks, Clustering, Gene expression, Time series, Dynamic network analysis, Dynamic data visualisation, Web application, Multi-conditional datasets, Background knowledge

## Abstract

**Background:**

Progress in high-throughput molecular methods accompanied by more complex experimental designs demands novel data visualisation solutions. To specifically answer the question which parts of the specifical biological system are responding in particular perturbation, integrative approach in which experimental data are superimposed on a prior knowledge network is shown to be advantageous.

**Results:**

We have developed DiNAR, Differential Network Analysis in R, a user-friendly application with dynamic visualisation that integrates multiple condition high-throughput data and extensive biological prior knowledge. Implemented differential network approach and embedded network analysis allow users to analyse condition-specific responses in the context of topology of interest (e.g. immune signalling network) and extract knowledge concerning patterns of signalling dynamics (i.e. rewiring in network structure between two or more biological conditions). We validated the usability of software on the *Arabidopsis thaliana* and *Solanum tuberosum* datasets, but it is set to handle any biological instances.

**Conclusions:**

DiNAR facilitates detection of network-rewiring events, gene prioritisation for future experimental design and allows capturing dynamics of complex biological system. The fully cross-platform Shiny App is hosted and freely available at https://nib-si.shinyapps.io/DiNAR. The most recent version of the source code is available at https://github.com/NIB-SI/DiNAR/ with a DOI 10.5281/zenodo.1230523 of the archived version in Zenodo.

**Electronic supplementary material:**

The online version of this article (10.1186/s13007-018-0345-0) contains supplementary material, which is available to authorized users.

## Background

Technological progress in biological data generation enhanced development of network modelling to allow comprehension at systems level [1]. The ideal *in silico* network should be concise and able to capture key features of the actual system. Although this is difficult to achieve, particularly with non-model organisms, network-based strategies have proven very useful for interpreting biological data [2]. In line with emerging network views of biological systems, development of user-friendly visualisation tools becomes even more relevant.

Efficient network visualisation is lagging behind, especially in exploration of multi-conditional setups. Few solutions combining background knowledge and network analysis to enable visualisation of experimental data have so far been implemented in this area [3–9]. We developed an application to extend existing tools and further facilitate biological insight into dynamic rewiring events. DiNAR uses prior knowledge accompanied by differential network analysis to visualise complex experimental datasets. Main advanced features of DiNAR are (1) dynamic visualisation of complex multi-conditional experiments, (2) identification of strong differential interactions and (3) recall of latent effects that are present in multi-conditional experiments. Although DiNAR was primarily set for analysis of *Arabidopsis thaliana* and *Solanum tuberosum* datasets, it can handle other background knowledge networks in combination with experimental dataset of interest, e.g. transcriptomics, proteomics, metabolomics.

## Implementation

DiNAR is written in R [10] and extended with JavaScript and Shiny package for interactive web applications. The implementation requires R version 3.1 or higher and several R packages, including animatoR [11], visNetwork [12] and ndtv [13]. Homotopy, as implemented in animatoR package, is used to interpolate node and edge weight value between discrete conditions/time-points. visNetwork, a browser based visualization library, allows implementation for easier manipulation and interaction with the data. Implementation of the ndtv package functions provides downloadable interactive movie rendering.

The fully cross-platform validated application is hosted and freely available at https://nib-si.shinyapps.io/DiNAR. Source code is stored at https://github.com/NIB-SI/DiNAR, where a more detailed application manual and package list are available. DiNAR can also be run locally in R or hosted on a local RStudio Shiny Server.

Current application release provides the user with two embedded background knowledge networks: manually constructed plant immune signalling network (PIS) [14] translated to *S. tuberosum* at the orthologue groups level [15] and one constructed from prior knowledge on *A. thaliana*—the *A. thaliana* Comprehensive Knowledge Network (AtCKN) [15]. AtCKN, containing 20,012 nodes and 70,091 connections, was first analysed to determine disjoint communities (i.e. clusters) based on network centrality measures, for easier visualisation. Multi-level community detections algorithm followed by spinglass community detection algorithm were used, both implemented in igraph R package [16]. As the result, AtCKN was divided into 48 clusters. DiNAR also provides an option of uploading a user-defined network. Any kind of network in the proper format can be used to visualise changes in omics dataset (e.g. transcriptomics, miRNAomics, proteomics and metabolomics). Notice that the background network node identifiers should be consistent with the corresponding experimental data identifiers as well as the statistical analyses between different omics levels have to be standardised. Both static graphics and interactive animations can be exported, together with a record of user settings, which is compliant with FAIR guiding principles for reproducible research [17].

In addition to DiNAR core scripts, optional pre-processing and clustering tools (subApps) are also hosted at shinyapps.io platform: https://nib-si.shinyapps.io/pre-processing and https://nib-si.shinyapps.io/clustering, while their source code is freely available under GitHub subApps sub-repository. Microarray/NGS data analysis from GEO with examples is available under GEODataAnalysis sub-repository. Gene orthologue groups of *A. thaliana*, potato and several other non-model plant species are available at GoMapMan web-accessible resource [18]. Test examples, description of clustering analysis pipeline, instructions for installation and technical troubleshooting guide are also deposited at https://github.com/NIB-SI/DiNAR/.

## Results and discussion

### Application features

#### DiNAR core app

**Fig. 1:**
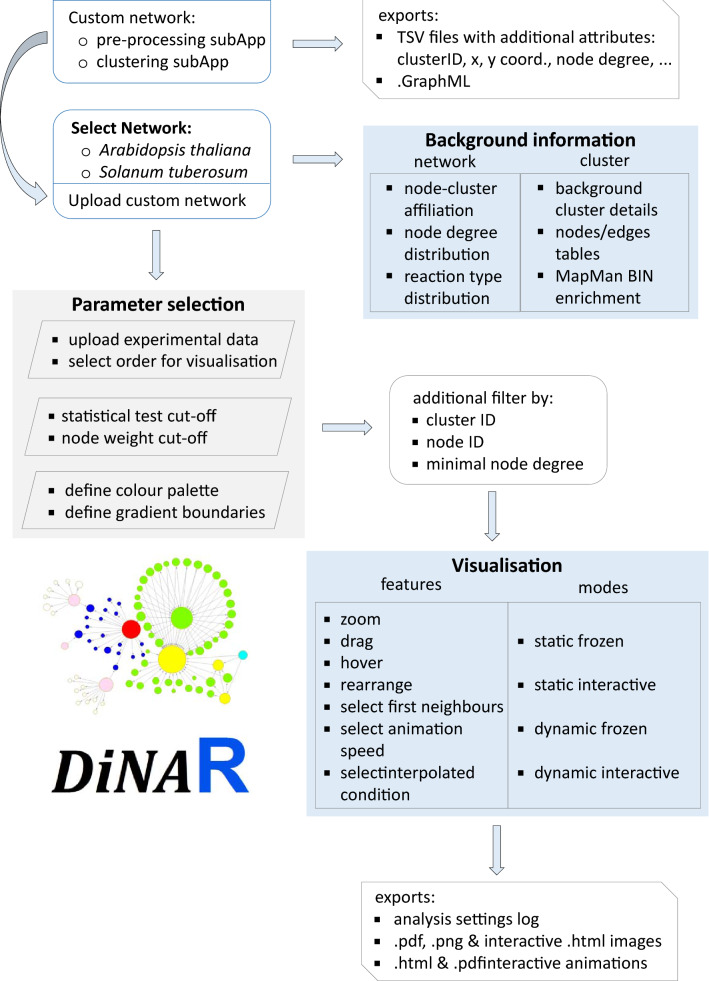
Overview of DiNAR features. DiNAR reads analysis-ready network and experimental data. It creates differential network per condition to produce interactive animation according to user-defined parameters

DiNAR landing web page provides the application overview, with links to user tutorials and tools. The interactive sidebar menu provides guidance through the required analysis steps (Fig. 1). In the case of any inconsistencies or missing steps, the application will not proceed to the next step.

For differential network visualisation, user selects among provided *A. thaliana* or *S. tuberosum* networks or uploads the pre-processed (preferably by DiNAR subApp) user-defined background knowledge network. DiNAR module size limit, in the term of visualisation, is between 2 and 16,384 edges, excluding loops (self-activation or self-inhibition, e.g. autocatalysis, oligomerisation, and autophosphorylation). Next, user uploads the experimental dataset of interest. Condition-specific networks are then dynamically constructed according to user-defined cut-off parameters: thresholds for the measure of statistical significance (e.g. adjusted p value < 0.05) and the threshold for the node weight values (e.g. interpolated absolute values of logFC $\geqslant$ 0.5, here denoted with $abs (\mathbf{n})$ and $abs (\mathbf{m})$). If two connected nodes (elements from **n** and **m**) pass the cut-off criteria, they and the edge connecting them are visualised over the selected background network. Node weight values are used to define the node size and colour as well as to define degree of edge weight $\left( \frac{\frac{abs(\mathbf {n})}{max(abs(\mathbf {n}), 1)}+\frac{abs(\mathbf {m})}{max(abs(\mathbf {m}), 1)}}{max(\frac{abs(\mathbf {n})}{max(abs(\mathbf {n}), 1)}+\frac{abs(\mathbf {m})}{max(abs(\mathbf {m}), 1)},1)}\right)$. As DiNAR does not use fixed cut-off thresholds, the user is allowed to fine-tune which level of expression change to disregard in a particular in silico investigation and to detect more subtle differences. If larger clusters are imported the user-friendly additional filtering by node degree is provided.

**Fig. 2:**
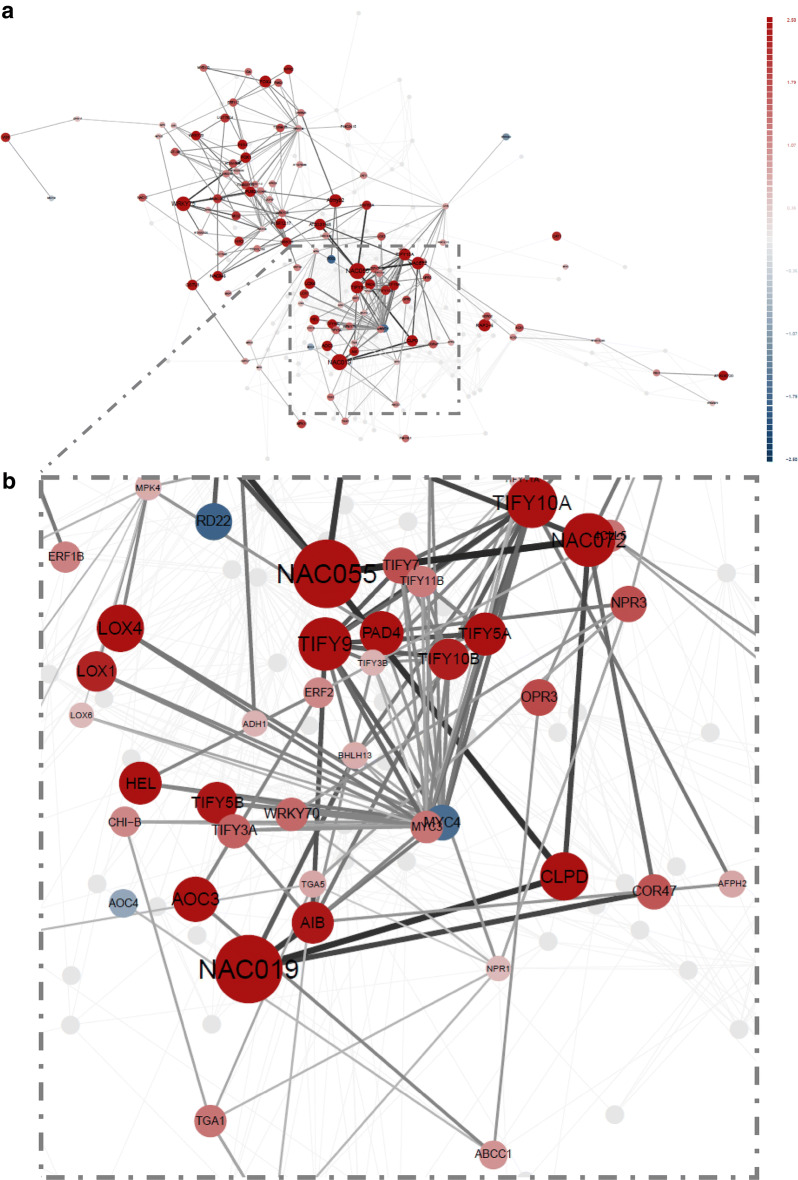
Arabidopsis thaliana response to bacterial pathogen *Pseudomonas syringae* DC3000. DiNAR static network visualization **a** of cluster 40 in the AtCKN in response to infection with *Pseudomonas syringae* 17.5 hpi (adjusted p value < 0.05, absolute value of logFC $\geqslant$ 0.5, min node degree 3). **b** Magnification of panel** a** as visible in dynamic-animatoR mode. Node colours correspond to gene regulation with red (upregulated) and blue (downregulated). Colour scale on top right. The size of nodes correspond to absolute logFC values

**Fig. 3:**
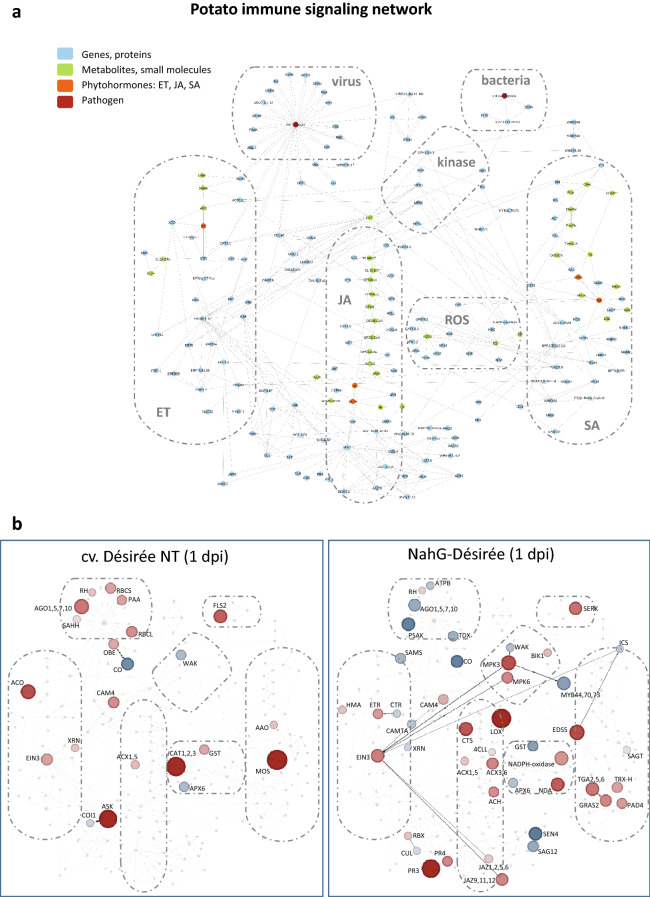
Visualisation of data in PIS network using DiNAR. **a** Potato immune signalling network comprising metabolic, signalling and gene regulatory pathways of SA, JA and ET. Nodes representing transcripts/proteins are coloured blue, metabolites and small compounds green and phytohormones orange. Pathogens are represented as red nodes. Parts of network describing SA, JA and ET pathways, kinases and ROS signalling as well as viral and bacterial interactors are marked with dashed line. **b** Transcriptional response of potato leaves to infection with PVY 1 dpi. DiNAR static–interactive visualisation of PIS network of NT cv. Désirée (left panel) and NahG-Désirée (right panel) is shown (adjusted p value < 0.05, no logFC cut-off, min node degree 1). Node colours correspond to gene regulation with red (upregulated) and blue (downregulated). The size of nodes correspond to absolute logFC values. Edges describing the reactions between the components are directed and represent activation (full line, unilateral arrows), inhibition (full line) or binding (dashed line, bilateral arrows)

Other panel tabs offer detailed information about the nodes and edges in the network, MapMan bin enrichment per cluster and two interactive network graphics views, static and dynamic. Options such as click and drag, zoom, hover and first neighbour highlight help users to explore rewiring events details. Static (background, frozen and interactive modes) and dynamic (vizNetwork and animatoR modes) results can be exported from the application, as well as the analysis settings log. See Fig. 1 for detailed overview of DiNAR features.

#### DiNAR subApps

Pre-processing subApp facilitates the construction of DiNAR$'$s Custom Network. Generally, nodes and edges tables with predefined structure for DiNAR input could be constructed manually (see the manual for more details), however for larger networks this would be unnecessarily time-consuming. Pre-processing subApp reads the supplied nodes and edges in three formats: tables (tab, comma or semicolon separated), GraphML (standard graph structure data format) or GraphML combined with XGMML. GraphML and XGMML formats can be exported from Cytoscape 3.6 [19], yEd [20] and similar applications/platforms. GraphML with node/edge attributes is also generated and could be exported from the subApp. In the case of uploading one of the first two formats, either a table or a sole GraphML file, coordinates are assigned to each node using a two-dimensional grid followed by Kamada–Kawai layout. Should the user want to use own defined coordinates, both the GraphML and XGMML files need to be uploaded. In addition, the pre-processing subApp also calculates the node degree measure. Files generated by the subApp are available at https://github.com/NIB-SI/DiNAR/tree/master/subApps/clustering/examples.

For the visualisation of large networks (e.g. AtCKN) to be informative, the network clustering step is necessary. Network of interest should be provided in GraphML format. Prior to the clustering step, the network is simplified by the removal of self-loops and duplicated edges. The first clustering step detects community structure using multi-level modularity optimization algorithm, excluding communities (i.e. clusters) with less than 5 nodes from further analyses. In the next step, densely connected (approximately fully connected subgraphs) and star-like clusters are identified. Densely connected clusters are defined as clusters with $n(n-1)$ edges, with n being the number of nodes. Star-like communities are defined as graphs that contained only one hub node and with an edge count close to the number of nodes. A hub node is defined as the node with a degree higher or equal to 60$\%$ of the maximal node degree of the cluster, high closeness, and betweenness in undirected graph close to $(n-1)(n-2)/2$, where *n* is the number of nodes. Upon identification of densely connected and star-like clusters, they are excluded from further processing, as are all clusters with less than $2^{10}$ nodes and $2^{11}$ edges. The remaining clusters are clustered further using spinglass community detection algorithm into the same number of sub-clusters as the number of hub nodes calculated for that cluster. For visualisation, first a two-dimensional grid layout is used, followed by either Fruchterman-Reingold (number of nodes $\le$ $2^6$ and number of edges $\le$ $2^6$ ) or Kamada–Kawai layout (otherwise). The final output of the clustering subApp are tables of nodes and edges, with information about cluster affiliation, node coordinates and degree, whilst preserving information about between cluster relations.

### Method validation

Overview of DiNAR features and most similar available applications is presented in Table 1. As no software was directly comparable to DiNAR, we validated our approach through biological interpretation of two plant pathogen interaction experimental datasets. Plant immune signalling response against pathogens is a complex phenomenon that involves plant perception of the pathogen, transduction of the signal within the plant cell and results in reprogramming of the plant metabolism [21]. Hormonal crosstalk, in general, plays an important role in plant responses to stress. In addition to ethylene (ET), jasmonic acid (JA) and salicylic acid (SA) [22], the importance of other plant hormones, whose basic function is not defence, including abscisic acid (ABA), gibberellin (GA), auxin (AUX) and cytokinin (CK) was shown. Depending on the pathogen and host physiological state, specific plant components are activated or repressed in a well-defined time-dependent manner. Analysing large datasets in DiNAR enables researchers to comprehend the regulatory events of such complex, inter-connected and highly dynamical biological systems.

**Table 1:** Comparison of DiNAR with most similar tools available

App name	App type	Type of visualisation	Network type import	Import of exp. datasets	Built-in clustering	Built-in filtering	Export formats	Technical operability
DiNAR	Stand-alone web app	Interactive: dynamic & Static	Preprocessed cytoscape, TSV text, ... (subApps)	*YES*	*YES* (DiNAR subApps clustering)	Stat. significance, node weight, min.node degree, cluster/gene ID	Interactive: dynamic & static; log, TSV, GraphML	*YES*
AIM [3]	Online	Static database	*NAp*	*NO*	*NAp*	*ND*	*ND*	*NO*
CyLineUp [4]	Cytoscape 3 plugin	Noninteractive static	Cytoscape	*YES*	*NO*	Stat. significance (p value)	Noninteractive static	*YES*
DyNet [5]	Cytoscape 3 plugin	Semi-interactive static	Condition specific	*NO*	*YES* (heatmap)	*ND*	Noninteractive static	*YES*
DyNetviewer [6]	Cytoscape 3 plugin	Coarse dynamic	text	*YES*	*YES*	Gene expression, SD	Text	*YES*
Diffany [7]	Cytoscape 3 plugin	Noninteractive static	Cytoscape	*NO*	*NO*	Edge weight	Noninteractive static	*YES*
PCSF [8]	R package	Interactive static	Edge list	*YES*	*YES*	Functional enrichment	Igraph	*YES*
iNID [9]	Online	Static	*NAp*	*YES*	*YES*	p values logFC	*ND*	*NO*

*exp.* experimental, *NAp* not applicable, *ND* not possible to determine, *SD* standard deviation, *technical operability* accesibility at the time of testing, *TSV* tab separated values

#### Arabidopsis thaliana

We first demonstrate DiNAR on a transcriptomics dataset describing dynamics of *A. thaliana* response to bacterial pathogen *Pseudomonas syringae* DC3000 at 0, 2, 3, 4, 6, 7, 8, 10, 11, 12, 14, 16 and 17.5 hours post inoculation (hpi) [23]. We interpreted Arabidopsis network cluster 40 (Fig. 2a), which includes several well-characterized immune-related genes. In the original analysis of the data by Lewis et al. [23], two notable peaks of global expression were observed, the first at 2 hpi and the second at 6 hpi. This is also evident in DiNAR dynamic visualisation of cluster 40, as transcriptional switches of two main gene modules (Additional file 1). The first switch corresponds to upregulation of a module containing hub transcription factors WRKY6/30 and ZAT11. In the second switch, another module containing key regulators of salicylic acid response TGA3/5, NPR3/4, PAD4 and EDS1 is activated. Furthermore, two additional transcriptional switches were identified with DiNAR visualisation. The first, occurring at 7 hpi, corresponds to induction of a module containing important jasmonate and ethylene response transcription factors and jasmonate biosynthesis genes. The second corresponds to reactivation of the previously mentioned salicylic acid response module at 17.5 hpi (Fig. 2b). These results illustrate how DiNAR provides easy insight into studied process and reveals additional information in comparison to using solely standard statistical analyses.

#### Potato-virus PVY interaction

For the second example we analysed potato time series transcriptional response to potato virus Y (PVY) [24] in the manually constructed plant immune signalling network [14] comprising of 205 nodes and 422 edges, covering metabolic, signalling and gene regulation networks of SA, JA and ET (Fig. 3a). The network was originally built by extensive article curation for Arabidopsis. Here we used its potato translation based on orthologue gene information [15]. In potato dataset, gene expression was measured 1, 3, 4, 5 and 7 days post inoculation (dpi). Two genotypes were analysed; non-transgenic (NT) cv. Désirée and its transgenic counter-part depleted in accumulation of salicylic acid (NahG-Désirée).

To evaluate the role of SA in potato immune signalling, dynamic gene expression changes between NT Désirée and NahG-Désirée were compared using Dynamic-VisNetwork and Dynamic-animatoR implemented in DiNAR (Additional files 2 and 3). The results show that SA-deficiency perturbs the transcriptional reprograming of the genes included in PIS network.

MAP kinases (MPKs) are regulated in different stages (1, 4 and 7 dpi) of NahG-Désirée response, even though no differences in expression of those genes were observed in NT plants. Interestingly, induction of MPK3 and MPK6 at 1 dpi coincides with the activation of the main ET responsive transcription factor EIN3 and respiratory burst enzyme NADPH-oxidase which expression levels are also induced. SA-deficiency changes the responsiveness of hormonal signalling pathway at different stages. For example, at 1 dpi components of JA biosynthesis (LOX, CTS, 4CLL, ACX3,6, and ACX 1, ACH) and JA signalling (JAZ 9, 11, 12 and JAZ 1, 2, 5, 6) are induced in NahG-Désirée, however remain unresponsive in NT genotype.

Even though SA is traditionally viewed as the most important hormone in virus-induced response [25], we show dynamic changes in expression of several ET-signalling components at different stages of Désirée NT response to PVY (Fig. 3b). At 1 dpi an induction of ACO (ACC oxidase), an ET-biosynthesis gene is observed and continuous to be induced at later time points (4 and 5 dpi), reaching the highest peak at 7 dpi. EIN3 is induced at 1 and 3 dpi and it induces the transcription of EBF gene resulting in expression peak of EBF gene at 3 dpi. At 4 dpi repression of several ethylene responsive factors (ERF1,2; ERF5,6; ERF 104, ERF105) as well as EBF gene occurs. With these examples, we show that DiNAR enables novel insights into dynamic gene expression reprogramming.

## Conclusion

The advantage of DiNAR, compared to other network visualization tools, is the dynamic visualization of multi-conditional datasets in the context of background knowledge on molecular interactions with embedded network analyses. It facilitates detection of network-rewiring events, gene prioritisation for future experimental design and allows capturing dynamics of complex biological system. Biological examples demonstrated how DiNAR provides valuable information in revealing hidden patterns of plant signalling dynamics and knowledge extraction.

## Additional files

**Additional file 1.** Dynamic visualisation of *Arabidopsis thaliana* response to *Pseudomonas syringae*. AtCKN network, cluster 40, GSE56094 experimental data, *Pseudomonas syringae* pv. tomato DC3000 vs Mock subset. Relative expression between *Pseudomonas syringae* and mock-treated plants has been log2 transformed. The absolute values are represented by the size of the node and differential expression is color-coded (red-induction, blue-repression of expression). Only genes that are significantly differentially expressed are visualized (FDR p < 0.05). Dynamic changes in gene expression following 0, 2, 3, 4, 6, 7, 8, 10, 11, 12, 14, 16 and 17.5 hpi are shown.

**Additional file 2.** Dynamic visualisation of immune signalling network response in potato cv. Désirée infected with virus PVY. PIS network, GSE58593 experimental data at the orthologue groups level. Relative expression between PVY and mock-treated plants has been log2 transformed. The absolute values are represented by the size of the node and differential expression is color-coded (red—induction, blue—repression of expression). Only genes that are significantly differentially expressed are visualized (FDR p < 0.05). Dynamic changes in gene expression 1, 3, 4, 5 and 7 dpi after infection with PVY are shown for Désirée plants.

**Additional file 3.** Dynamic visualisation of immune signalling network response in potato cv. NahG-D\'esir\'ee infected with virus PVY. PIS network, GSE58593 experimental data at the orthologue groups level. Relative expression between PVY and mock-treated plants has been log2 transformed. The absolute values are represented by the size of the node and differential expression is color-coded (red—induction, blue—repression of expression). Only genes that are significantly differentially expressed are visualized (FDR p < 0.05). Dynamic changes in gene expression 1, 3, 4, 5 and 7 dpi after infection with PVY are shown for NahG-Désirée plants.

## Abbreviations

Ath: *Arabidopsis thaliana*
app: application
CKN: Comprehensive Knowledge Network
dpi: days post inoculation
exp.: experimental hpi hours post inoculation
ID: identifier
MPKs: MAP kinases
min.: minimal
NA: not available
NAp: not applicable
ND: not determined
NT: non-transgenic
PIS: plant immune signalling
SD: standard deviation
stat.: statistical
subApp: subApplication
tp: time point
