# Supplementary material for: DiNAR: revealing hidden patterns of plant signalling dynamics using Differential Network Analysis in R
Source: Plant Methods. 2018 Aug 30;14:78. doi: 10.1186/s13007-018-0345-0 (PMC6117943; doi:10.1186/s13007-018-0345-0)

Differential Network Analysis in R

Arabidopsis thaliana CKN

Experimental data: GSE56094

Subset: *Pseudomonas syringae* pv. tomato DC3000 vs Mock

Cluster: 40 [minDegree: 3]

n: 211 e: 1033

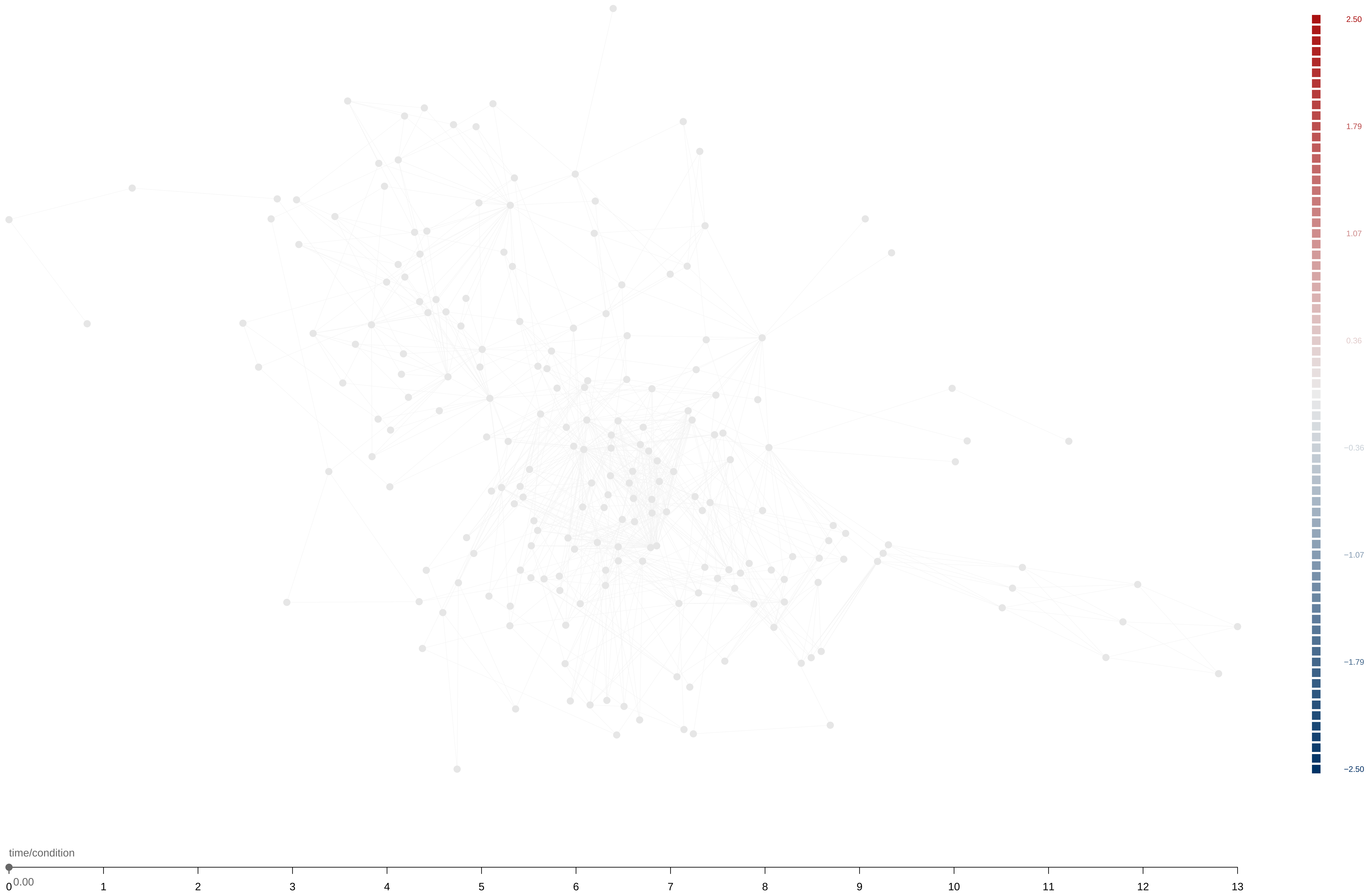

Supplement: Supplementary file 1 — Additional file 1. Dynamic visualisation of Arabidopsis thaliana response to Pseudomonas syringae. AtCKN network, cluster 40, GSE56094 experimental data, Pseudomonas syringae pv. tomato DC3000 vs Mock subset. Relative expression between Pseudomonas syringae and mock-treated plants has been log2 transformed. The absolute values are represented by the size of the node and differential expression is color-coded (red-induction, blue-repression of expression). Only genes that are significantly differentially expressed are visualized (FDR p < 0.05). Dynamic changes in gene expression following 0, 2, 3, 4, 6, 7, 8, 10, 11, 12, 14, 16 and 17.5 hpi are shown. [file 13007_2018_345_MOESM1_ESM.pdf]
