# Supplementary material for: DiNAR: revealing hidden patterns of plant signalling dynamics using Differential Network Analysis in R
Source: Plant Methods. 2018 Aug 30;14:78. doi: 10.1186/s13007-018-0345-0 (PMC6117943; doi:10.1186/s13007-018-0345-0)

Differential Network Analysis in R

Solanum tuberosum Immune signalling

Experimental data: GSE58593

Subset: Désirée PVY infected vs Mock

Cluster: 1 [minDegree: 1]
n: 205 e: 422

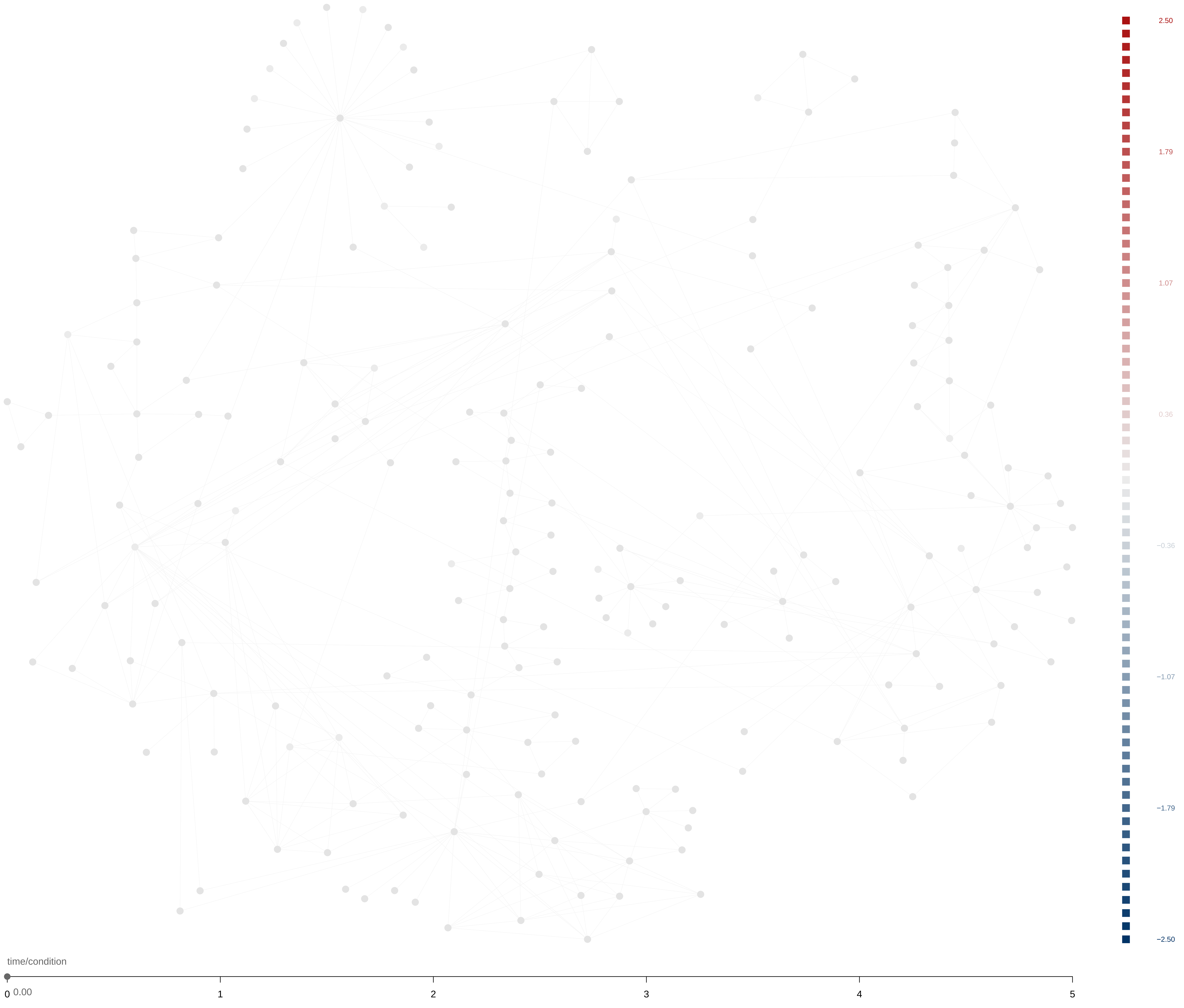

Supplement: Supplementary file 2 — Additional file 2. Dynamic visualisation of immune signalling network response in potato cv. Désirée infected with virus PVY. PIS network, GSE58593 experimental data at the orthologue groups level. Relative expression between PVY and mock-treated plants has been log2 transformed. The absolute values are represented by the size of the node and differential expression is color-coded (red—induction, blue—repression of expression). Only genes that are significantly differentially expressed are visualized (FDR p < 0.05). Dynamic changes in gene expression 1, 3, 4, 5 and 7 dpi after infection with PVY are shown for Désirée plants. [file 13007_2018_345_MOESM2_ESM.pdf]
