# Supplementary material for: DiNAR: revealing hidden patterns of plant signalling dynamics using Differential Network Analysis in R
Source: Plant Methods. 2018 Aug 30;14:78. doi: 10.1186/s13007-018-0345-0 (PMC6117943; doi:10.1186/s13007-018-0345-0)

Differential Network Analysis in R

Solanum tuberosum Immune signalling

Experimental data: GSE58593

Subset: NahG−Désirée PVY infected vs Mock

Cluster: 1 [minDegree: 0]

n: 205 e: 422

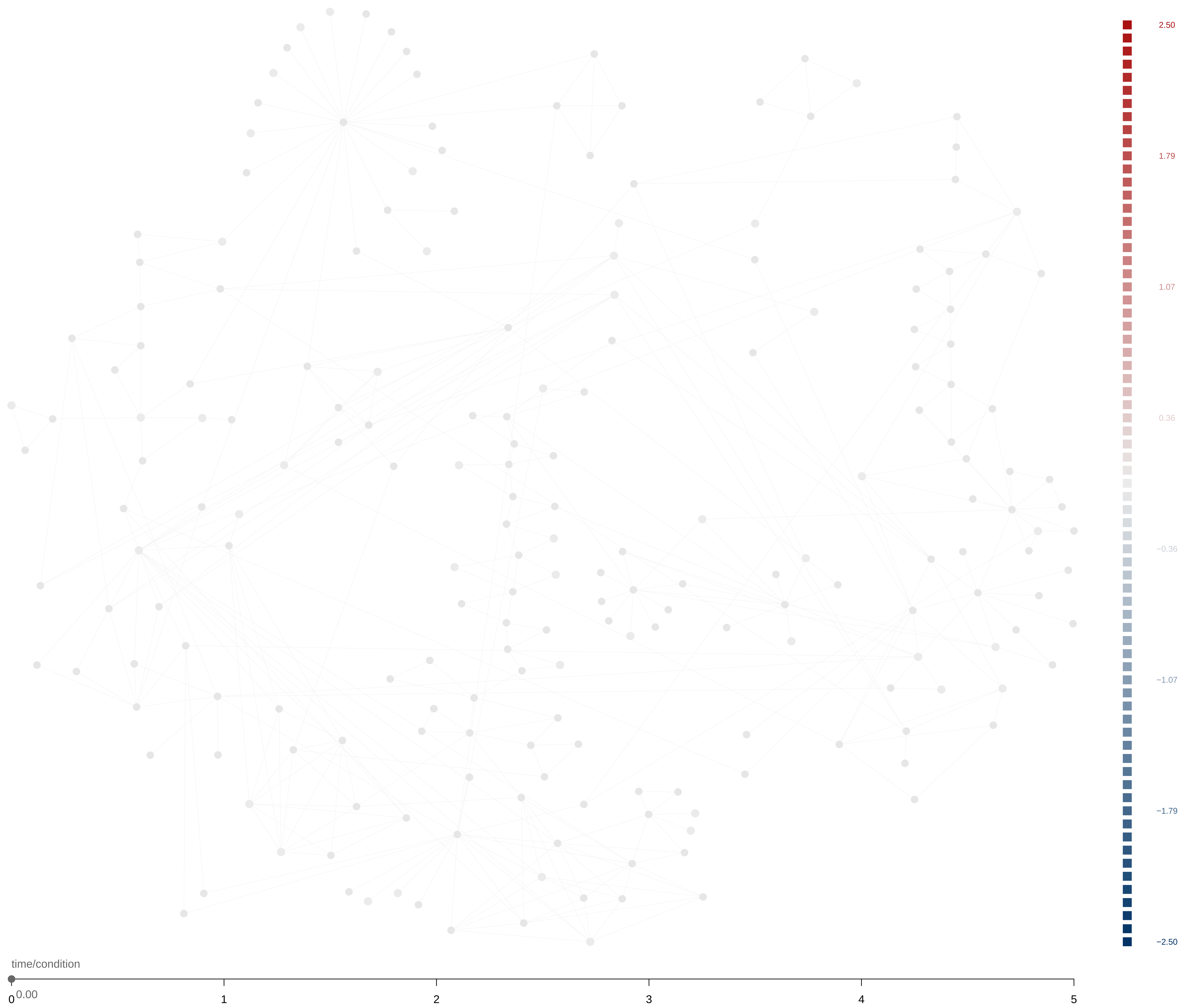

Supplement: Supplementary file 3 — Additional file 3. Dynamic visualisation of immune signalling network response in potato cv. NahG-D\'esir\'ee infected with virus PVY. PIS network, GSE58593 experimental data at the orthologue groups level. Relative expression between PVY and mock-treated plants has been log2 transformed. The absolute values are represented by the size of the node and differential expression is color-coded (red—induction, blue—repression of expression). Only genes that are significantly differentially expressed are visualized (FDR p < 0.05). Dynamic changes in gene expression 1, 3, 4, 5 and 7 dpi after infection with PVY are shown for NahG-Désirée plants. [file 13007_2018_345_MOESM3_ESM.pdf]
